# Role of Traditional Cardiovascular Risk Factors after Initiation of Statin Therapy: A PharmLines Inception Cohort Study

**DOI:** 10.1155/2022/6587165

**Published:** 2022-05-24

**Authors:** Dennis Steenhuis, Stijn de Vos, Jens Bos, Eelko Hak

**Affiliations:** Department of Pharmacotherapy, Epidemiology, and Economics, University of Groningen, Groningen Research Institute of Pharmacy, Groningen, Netherlands

## Abstract

**Background:**

Multiple studies and meta-analyses examined the role of traditional risk factors for cardiovascular events in statin treatment-naive patients. Nowadays, millions receive such therapy for the primary prevention of cardiovascular events (CVE).

**Objective:**

CVEs still occur in patients on primary preventive statin therapy. Therefore, further risk stratification within these patients is urgently needed.

**Methods:**

Using the unique linkage between biomedical data and prescription data from the PharmLines Initiative, we assessed the role of several risk factors used in cardiovascular risk models, using a time-dependent Cox PH model, in the occurrence of drug treatment of CVEs after initiation of statin therapy.

**Results:**

Among 602 statin therapy starters, 11% received drug treatment for CVE within an average follow-up period of 832 days. After multivariable modelling, cholesterol levels and blood pressure at baseline were no longer associated, whereas self-reported diabetes and increasing age were highly associated with the outcome when on statin therapy (hazard ratio (HR): 3.01, 95% confidence interval (95% CI): 1.48-6.12 and 1.04; 95% CI: 1.01-1.07, respectively). Males, smokers, and nonadherent patients had increased risks (HR 1.6, 1.12, and 1.18, resp.), though not statistically significant.

**Conclusion:**

Drug treatment for CVEs after statin initiation is increased in patients with diabetes type 2, in aged patients, males, smokers, and those with poor adherence, while there was no association with baseline cholesterol levels and blood pressure. These factors should be taken into account during the monitoring of statin therapy and may lead to changes in statin treatment or risk-related lifestyle factors.

## 1. Introduction

In 2019, approximately two million (12%) Dutch inhabitants were on lipid-lowering therapies, with the vast majority on 3-hydroxy-3-methylglutaryl-coenzyme A (HMG-CoA) reductase inhibitors or statins (97%) [1]. According to large-scale randomized controlled trials, statin therapy reduces the risk of major cardio- and cerebrovascular events (CVE) such as myocardial infarction and stroke in both primary and secondary prevention [2, 3]. In the Netherlands, according to the Dutch College of General Practitioners (NHG) guidelines, patients should receive statin therapy if the risk of experiencing a CVE in the next 10 years is more than 5% and/or if any of the following risk diseases is present: CVE, diabetes type 2, or chronic kidney failure [4]. The risk estimation for CVE is based on the Systematic Coronary Risk Evaluation (SCORE) algorithm developed by the European Society of Cardiology (ESC) [5].

Statins have been shown to reduce the LDL-C levels [6]; subsequently, reducing LDL-C levels lowers the risk of any major cardiovascular event [2, 3]. Even among the high-risk group of persons aged 75 years or older, statin therapy is beneficial in preventing cardiovascular events (CVEs) [7]. However, in these trials, an estimated 2.5% of the patients on statin therapy for primary prevention still developed a CVE within one to five years [3], and such rates are understudied in real-world settings. To further reduce the risks of CVEs in primary prevention practice, a more personalized approach is therefore warranted.

Traditionally, multiple studies and meta-analyses were carried out on risk factors of CVEs in treatment-naive patients without statin treatment [5, 8–11]. The derived risk score models, such as the European cardiovascular risk score [5], the Framingham risk score (FRS) [11], and the Reynolds risk score (RRS) [8, 9], are mainly based on age, gender, blood pressure, smoking status, and cholesterol levels from untreated patients. All of these algorithms perform similarly adequate in determining the risk in large-sized untreated populations, and therefore, such scores accurately guide current cardiovascular preventive actions [12].

Despite accurate models and proven statin effectiveness, in practice, statin nonadherence may reduce its impact. It has been shown that high statin adherence (>80%) decreases the risk of a CVD by 15 to 25% [13–15], but lower rates may have no effect.

In this paper, using the unique linkage between biomedical data and prescription data from the PharmLines Initiative [16], we assessed the associations of several risk factors as used in the score models on the risk of cardiovascular events in statin initiators. We did this to determine whether such factors are still modifying the risk of cardiovascular events in patients on primary preventive statin therapy, taking the dosing and adherence to statin therapy into account.

## 2. Methods

### 2.1. Setting and Data Source

In 2017, the Groningen Research Institute of Pharmacy (GRIP) started the PharmLines Initiative together with Lifelines to facilitate research on medical drug data in combination with the health and biobank data of the Lifelines cohort [16, 17]. The two databases are linked at a patient level by Statistics Netherlands (Centraal Bureau voor de statistiek (CBS)), which acts as a trusted third party (TTP) [18]. In 2018, about 60,000 Lifeliners could be uniquely linked and were available for research.

“The University of Groningen IADB.nl pharmacy prescription database is a growing database that contains prescription data for more than 20 years from 1996 till 2017 from approximately 70 community pharmacies and covers an estimated population of 700,000 patients. Registration in the database is irrespective of health care insurance and age, gender and prescription rates among the database population have been found to be representative of the Netherlands as a whole, and the database has been widely used for research. Each person is individually tracked throughout the database period and prescription records contain information on the date of dispensing, the quantity dispensed, the dose regimen, the number of days the prescription is valid, the prescribing physician and the Anatomical Therapeutic Chemical code (ATC code). Each patient has a unique anonymous identifier; date of birth and gender are known. Due to the high patient-pharmacy commitment in the Netherlands, the medication records for each patient are virtually complete, except for over the counter (OTC) drugs and medication dispensed during hospitalization.” [17, 19–21].

“The linkage is made with the Lifelines database, which started in 2006. Lifelines is a multi-disciplinary prospective population-based cohort study examining in a unique three-generation design the health and health-related behaviors of 167,729 persons living in the North of The Netherlands. It employs a broad range of investigative procedures in assessing the biomedical, socio-demographic, behavioral, physical and psychological factors which contribute to the health and disease of the general population, with a special focus on multi-morbidity and complex genetics. Participants are asked to complete a questionnaire every 1.5 years and visit a Lifelines location where several measurements and test are conducted and biological samples are collected every 5 years. The questionnaire data includes for example lifestyle and health questions, while the measurements, tests and biological samples include BMI, blood pressure and blood measurements.” [18, 21–23].

### 2.2. Study Population

The study population consisted of PharmLines participants over 20 years of age who initiated statin therapy recommended by a general practitioner or physician based on the guidelines of the NHG [4] for primary prevention of cardiovascular events anywhere between 2006 and 2017.

### 2.3. Inclusion and Exclusion Criteria

We selected those patients in the PharmLines Initiative who were 20 years or older at the date of their first statin prescription (index date), who had at least one statin prescription within a year after the index date, and who had at least two years of history in the IADB.nl database before starting statin treatment. We excluded patients who did not visit a Lifelines location within two years before the index date to prevent information bias.

To select primary prevention, we selected patients free of cardio- or cerebrovascular events at baseline and patients who had a recorded prescription of any of the following medications used in the acute treatment of cardiovascular events: vitamin K antagonists with the Anatomical Therapeutic Classification (ATC)-code B01AA, platelet aggregation inhibitors (B01AC), organic nitrates (C01DA), or other vasodilators used in cardiac diseases (C01DX) [24, 25] in two years before or within 90 days after the index date were excluded. The abovementioned four drug classes were selected based on a validation study examining the validity of applying these proxy medications for the exclusion of existing cardiovascular disease patients using a pharmacy-dispensing database [26]. These four proxy medications identified a history of major ischemic heart disease (IHD) or cerebrovascular disease diagnosed by a general practitioner (GP) or in a hospital with a sensitivity of 85% and specificity of 75%. In addition to these drug verifications, patients who reported any history of cardiovascular events in the Lifelines questionnaire by answering “yes” on one of the questions “Have you ever had a stroke?”, “Have you ever been diagnosed with a narrowing in one or both carotid arteries?”, or “Have you ever had a balloon angioplasty (stretching of artery with balloon) and/or bypass surgery?” were also excluded.

### 2.4. Outcome

The outcome drug treatment for a CVE was defined on the basis of the initiation of at least one prescription of the abovementioned drugs vitamin K antagonists with the Anatomical Therapeutic Classification (ATC)-code B01AA, platelet aggregation inhibitors (B01AC), organic nitrates (C01DA), or other vasodilators used in cardiac diseases (C01DX) [24, 25] whichever came first. The validation study showed that indeed 57% of patients with an encoded incident GP or hospital diagnosis of major IHD or cerebrovascular event were treated with such medications [26]. Importantly, the negative predictive value was as high as 94% which means that only 6% without the acute cardiovascular medications should have been classified as a patient with a CVE. Time-to-event is defined as the time from the first statin prescription (index date) until the first prescription of any of these drugs. Patients who left the pharmacy in the IADB.nl database or reached the end of the study were censored.

### 2.5. Covariates

Information on age and sex was collected at the index date. Information on other covariates such as cholesterol levels (LDL-C, HDL-C, TC, and TG), blood pressure (diastolic and systolic), self-reported diabetes, and self-reported smoking status was collected not more than two years before the index date, via the Lifelines questionnaire or visit at a lifelines location. We defined hypertension as an in-office systolic blood pressure ≥ 140 mmHg and/or diastolic blood pressure ≥ 90 mmHg (grade 1 hypertension or higher) [27], independent of antihypertensive drug treatment or any other cardiovascular risk factors. Adherence to statin drug therapy was measured by combining the dispensing dates and amount of dispensed medication using the continuous, multiple interval measure of medication acquisition (CMA) [28], calculated over a period the same statin and dose were used. The statin adherence was categorized into two classes: (1) high adherence, more than 80%, and (2) low adherence, 0-80%. We further developed an equivalent doses (EQD) scheme of different statins such that the dose lowers the LDL-C in percentage points about the same [6, 29–31]. We categorized these EQD's into three classes: less than 30%, 30-45%, and more than 45% (see Table 1). Participants were defined as having discontinued statin medication or being nonpersistent, using a standard permissible gap model, when the prescription gap exceeds three times the dispensed daily supply of the last prescription [32]. The last three covariates, statin type, EQD, and adherence, were treated as time-dependent and were changed whenever a participant changed it statin type and/or dosage. Although we did not have accurate data on the initiator of changes in statin type and/or dose, this is most likely the patient's general practitioner. In the Netherlands, almost all citizens are registered with on the GP practice.

### 2.6. Statistical Analysis

Patients with or without outcome were compared using the *χ*^2^-test for discrete variables, to test for different relative frequencies in subgroups and independent-sample Welch's *t*-test for normally distributed continuous variables, to test for differences in the mean. The Kaplan-Meier curves were constructed for the subgroups according to sex, hypertension, and self-reported diabetes, as the main categorical risk factors of cardiovascular events in non-statin users. After construction, the log-rank test was used to compare these survival curves. For comparison, we started with fitting univariable time-dependent Cox models to determine the univariable hazard ratios. Further, we fitted a time-dependent regularized Cox proportional hazard's model with elastic net penalty [33] from the first statin prescription until a CVE or censoring to determine multivariable hazard ratios. We used a time-dependent regularized Cox model instead of the standard time-dependent Cox model because of the relatively low number of observations per variable. The dataset was split up in a training and test set, with a ratio of 70-30%, to determine the best combination of the elastic net penalty hyperparameters over a 2-dimensional grid of 189 different combinations [33]. The best combination of parameters was defined as the one with the lowest partial likelihood deviance based on a tenfold cross-validation on the training set. Finally, a time-dependent regularized Cox model was fit on the training set to determine the hazard ratios. The 95% confidence intervals around the non-unity hazard ratios are estimated using the inverse of the observed Fisher information matrix [34, 35]. A *p*-value of 0.05 is considered statistically significant for all performed tests. To validate the fitted time-dependent regularized Cox model, time-dependent AUC scores are calculated according to a method with cumulative sensitivity and dynamic specificity (C/D) described as method CD4 by Kamarudin et al. [36] on the test set. This AUC score is an aggregate measure of performance for the Cox model and shows the discriminating power of the model. The corresponding confidence interval is calculated using the method described by Hanley and McNeil [37]. Statistical analysis was done in R version 3.6.2 [38], with the use of the R packages survival and coxphw. The R code used to determine the multivariable regularized hazard ratios (regularized Cox model, with elastic net penalty) and implementation of the AUC calculation method can be found in the Supplementary data. Other codes are available on request via the first author.

## 3. Results

### 3.1. Study Population Characteristics

**Figure 1:**
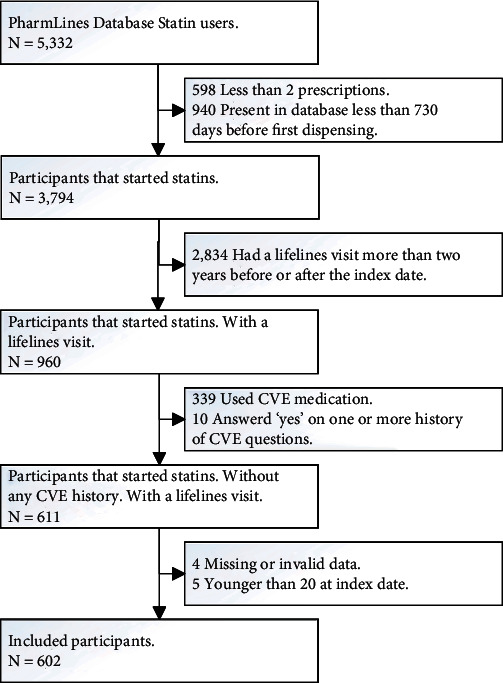
Scheme of exclusion route. Abbreviations: CVE: cardiovascular event.

In the PharmLines dataset, 5332 cohort members were on statin therapy during the study period. After applying strict in- and exclusion criteria, 602 patients without cardiovascular disease who started statin therapy were included in this study (see Figure 1).

From the 602, 66 (11.0%) received cardiovascular drug treatment with a mean time-to-event of 832 (SD: 546) days after the index date. The other 536 patients had a mean follow-up time of 1464 (SD: 765) days. The included patients have a mean age of 56 (SD: 11) years, and 57% were women. The distribution of other characteristics according to outcome is shown in Table 2. Except for the presence of diabetes and mean cardiovascular risk score, no significant differences between the group with and without cardiovascular drug treatment could be found. The prevalence of diabetes was more than twice as high in the cardiovascular drug treatment group (18% vs. 7%) than in those without such an event. Also, the risk score in percentages of having a cardiovascular event within ten years, calculated using the European score algorithm, was higher in the cardiovascular drug treatment group (3.92% vs. 2.71%).

Patients who are non-adherent almost directly after initiating statin therapy show a statistically non-significant higher risk of receiving cardiovascular drug treatment (53% vs. 41.6%, Table 2). Approximately 60% of the primary preventive statin users are still taking statins three or more years after the index date. About 20% of the participants discontinued their treatment in the first year. With another 9% and 7% in the second and third year, respectively (see Table 3). Note that the total percentage of statin users is not always decreasing over time, since only participants that are still in the study at a specific time point are counted. Besides, participants could reinitiate statin therapy again, after they stopped for a while.

**Figure 2:**
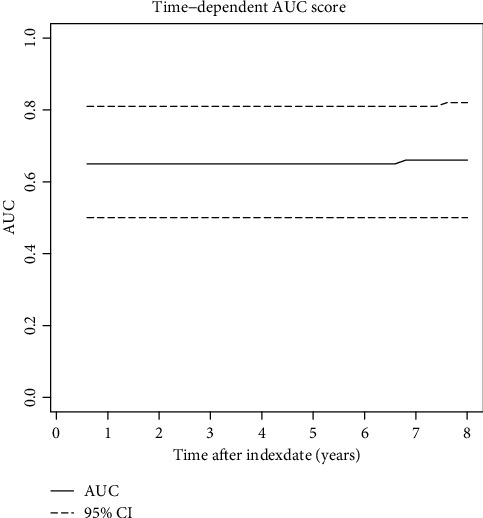
Time-dependent AUC scores on the test set of the regularized Cox model until 8 years after the index date. Abbreviations: AUC: area under curve; CI: confidence interval.

In the regularized Cox model, most hazard ratios were estimated to be non-significant and close to 1.0 (see Table 4). The presence of self-reported diabetes was highly associated with the risk of a drug-treated cardiovascular event when on statin therapy (hazard ratio (HR): 3.01, 95% confidence interval (95% CI): 1.48-6.12). Although there were trends towards increased risks for men, smokers, and those on atorvastatin and low adherence, statistical significance was not reached. All other risk factors showed no association, or HRs close to 1.0. Furthermore, the time-dependent AUC score of the test set had a mean of 0.65 (95% CI: 0.5-0.82) (see Figure 2).

## 4. Discussion

In this real-world population of statin starters, one in ten received drug treatment for CVEs after on average two and a half years. Traditional risk factors such as cholesterol levels or blood pressure for CVEs used in score models appeared to be no longer associated with these events once patients started using statins for primary prevention. However, patients with diabetes remained at three times higher risk for CVE, which is also observed in an untreated population with high cholesterol levels [11, 39] and in a recent study among women [40]. Also, increasing age was highly significantly associated with these CVEs with similar point estimates as in treatment naïve patients [11, 39, 40].

Though males ran higher risks, though non-significantly, for CVD treatment, the point estimates of the HRs were approximately the same as found in non-statin users by Wilson et al. [11] and Nakazato et al. [39]. Smokers had a slightly higher risk of suffering from a cardiovascular event than former or non-smokers. This agrees with previous research that shows that smoking increases the risk of cardiovascular events in comparison to non-smokers [1, 5, 8, 9, 11].

The fact that cholesterol levels and blood pressure at baseline do not play a role once patients start primary preventive statin treatment indicates that statin therapy influences cholesterol levels as well as blood pressure in such a way that the measurements at baseline do not further predict future cardiovascular events. This agrees with research that shows that statins reduce LDL-C [2] and have a positive, albeit small, effect on blood pressure [41, 42].

Since in both primary preventive statin users as well as non-statin users the risk of patients with diabetes remains considerable [11, 39], recent research among women shows that such risks are even much higher (range HRs from 3.5 to 10.7) in non-statin users depending on age [40]. This would indicate that risks do not decrease after initiation of statin therapy, and therefore, these patients warrant close monitoring and probably more intensified statin and/or diabetes treatment. Further research is needed on the application of modified scoring models for diabetes patients such as the Steno Type 1 Risk Engine (ST1RE) [43] for diabetes type 2 patients.

Increasing age also poses a risk, despite statin treatment. Given that most Western populations are ageing, older patients should be closely watched. Recently, Orkaby et al. [7] published on the potential benefits of statins in older people 75 years and over and showed an average 8% risk reduction within six years among this vulnerable population if treated as compared to non-treatment. Also, men show a higher risk compared to women, albeit non-significant. This coincides with the research of Irawati et al. [44] who showed that a similar dose and adherence rates, women appear to have a better response to statins compared to men.

Finally, we observed that approximately half of the patients did not fully adhere to their treatment. Importantly, we noted a decrease in participants who continued their primary preventive statin treatment from 100% to about 60% within three years. The statin discontinuation rates that were found in this research and summarized in Table 3 agree with earlier evidence from Alfian et al. [45], who examined diabetes type 2 patients who started statin therapy in the IADB.nl database (*N* = 12,741). Moreover, we noticed that high adherence (>80%) decreases the risk of cardiovascular drug treatment by about 18%. This coincides with the results found in earlier research [13–15].

### 4.1. Strengths and Limitations

A potential strength of this study is that it is set in an unselected population of statin initiators for primary prevention using real-world data, and therefore, the results are generalizable to such patient populations [46]. Importantly, since statin use was similar in a much larger cohort from the same setting (see Alfian et al. [45]), this implies no selection bias because of the strict in- and exclusion criteria in our study. Furthermore, we used a regularized Cox model instead of a standard Cox model to account for high variance and possible correlation between covariates [47]. In addition, we used time-dependent statin type, EQD, and adherence to account for patients that changed their prescription regime.

The outcome used in this study is cardiovascular drug treatment as this permitted us to have more accurate data on the timing of these events, and not all future events are already recorded as part of the third measurement in the Lifelines database which runs from 2019. As shown by an earlier validation study, not all patients receiving such treatment will have had a myocardial infarction or stroke, and some of those who ended up in the hospital without consultation with the GP and pharmacist will have been missed.

The time-dependent AUC score showed a mean of 0.65 on the test set, which indicates that the regularized Cox model demonstrated moderate discrimination for the outcome in statin initiators.

In our study, one in ten patients were drug treated for CVEs in on average 2.4 years. This is four times higher than the event rate of 2.5% found in a meta-analysis [3] and about three times higher than the event rates found by two other meta-analyses [48, 49]. Explanations could be that the trial populations are not fully representative of the real-world population and were for example younger and had no diabetes. Also, trials have strict treatment protocols where adherence rates are optimized. Further, as indicated, more events may have happened due to the definition of the outcome event. Higher event rates could also be an effect of LDL-C targets not being reached in a real-world setting. This is shown by Presta et al. [50], who concluded that in a real-world setting, LDL-C targets are not reached, despite being on cholesterol-lowering therapy and having (very) high-risk scores.

Counterintuitively, discontinuing statin treatment was not associated with an increased risk of cardiovascular drug treatment. Stopping primary preventive statin treatment may be recommended by a GP because of other external factors such as polypharmacy, a certain type of diets, or increased physical exercise [51, 52]. We could not take these modifying external factors into account in this research.

## 5. Conclusion

Drug treatment for CVEs still occurs in patients on primary preventive statin therapy. Cardiovascular drug treatment is more likely to occur in those who also suffer from diabetes type 2, in aged patients, males, smokers, and those with poor adherence. Baseline cholesterol levels and blood pressure did not seem to be associated with cardiovascular drug treatment. All these mentioned factors should be taken into account during monitoring of statin therapy and may lead to changes in statin therapy or risk-related lifestyle factors.

## Figures and Tables

**Table 1:** Equalized statins doses. Doses of statins are combined such that they have approximately the same effect on lowering LDL-C.

EQD	Low^a^		Medium^a^			High^a^		
Atorvastatin			10 mg	20 mg		40 mg	80 mg	
Atorvastatin + 10 mg ezetimibe			5 mg			10 mg	20 mg	40 mg
Cerivastatin	0.1 mg	0.2 mg	0.4 mg	0.8 mg				
Fluvastatin	20 mg	40 mg	80 mg					
Lovastatin	10 mg	20 mg	40 mg	80 mg				
Pitavastatin			1 mg	2 mg		4 mg		
Pravastatin	10 mg	20 mg	40 mg	80 mg				
Simvastatin	10 mg		20 mg	40 mg	60 mg	80 mg		
Simvastatin + 10 mg ezetimibe			10 mg			20 mg	40 mg	80 mg
Rosuvastatin			5 mg			10 mg	20 mg	40 mg

Notes: ^a^Low lowers the LDL-C 0-30%, Medium lowers the LDL-C 30-45% and high more than 45% [6, 28–30].

**Table 2:** Baseline characteristics of participants with and without cardiovascular drug treatment.

Variable	No cardiovascular drug treatment (*N* = 536)	Cardiovascular drug treatment (*N* = 66)	*p* value
Age, mean (SD), years	55.58 (10.9)	57.95 (10.63)	0.092
Sex, men	226 (42.2%)	34 (51.5%)	0.188
Smoking status			0.495
Yes	105 (19.6%)	17 (25.8%)	
Former	260 (48.5%)	29 (43.9%)	
Cholesterol, mean (SD), mmol/L	6.38 (1.25)	6.37 (1.12)	0.927
HDL-C, mean (SD), mmol/L	1.39 (0.45)	1.37 (0.43)	0.858
LDL-C, mean (SD), mmol/L	4.41 (1.18)	4.39 (1.03)	0.888
Triglycerides, mean (SD), mmol/L	1.97 (1.5)	1.91 (1.03)	0.734
Diastolic blood pressure, mean (SD), mmHg	77.44 (9.62)	79.85 (11.05)	0.094
Systolic blood pressure, mean (SD), mmHg	134.66 (17.44)	136.48 (19.66)	0.473
Hypertension, yes	36.6%	42.4%	0.427
Self-reported diabetes, yes	39 (7.3%)	12 (18.2%)	0.006
Familial CVD			0.036
Yes	93 (17.4%)	5 (7.6%)	
Do not know/missing value	393 (73.3%)	58 (87.9%)	
Cardiovascular risk score	2.71 (3.42)	3.92 (4.63)	0.043
Statins			0.135
Atorvastatin	42 (7.6%)	2 (4.5%)	
Rosuvastatin	31 (5.9%)	0 (0%)	
Simvastatin	455 (84.9%)	61 (92.4%)	
Other statin	9 (1.7%)	2 (3.0%)	
Dose^a^			0.341
Medium	399 (63.2%)	43 (65.2%)	
High	31 (5.8%)	1 (1.5%)	
Adherence			0.077
Low (0-80%)	223 (41.6%)	35 (53%)	

Notes: ^a^See Table 1 for grouping of doses. Abbreviations: CVD: cardiovascular disease; CVE: cardiovascular event; H(L)DL-C: high- (low-) density lipoprotein cholesterol; SD: standard deviation.

**Table 3:** Percentage statin users per year after index date.

Years after index date	0	1	2	3	4	5	6	7
Atorvastatin	7.3%	12.1%	11.4%	10.2%	8.1%	8.0%	9.0%	17.2%
Other statin	1.8%	2.4%	4.1%	4.7%	5.6%	4.3%	3.0%	3.4%
Rosuvastatin	5.1%	6.5%	8.2%	9.3%	6.9%	8.6%	9.0%	8.6%
Simvastatin	85.7%	58.2%	47.4%	40.4%	38.3%	35.6%	38.0%	34.5%
Total	100%	79.9%	71.1%	64.6%	59.9%	56.5%	59%	63.7%

**Table 4:** Hazard ratios for receiving cardiovascular drug treatment from univariable Cox regression and multivariable regularized Cox regression.

	Univariable		Multivariable	
	HR (95% CI)	*p* value	HR (95% CI)	*p* value
Men	1.63 (0.94-2.84)	0.083	1.6 (0.91-2.81)	0.103
Age, years	1.04 (1.01-1.07)	0.003	1.04 (1.01-1.07)	0.008
Cholesterol, mmol/L	0.91 (0.73-1.13)	0.391	1	—
LDL-C, mmol/L	0.92 (0.73-1.15)	0.465	1	—
HDL-C, mmol/L	0.87 (0.45-1.69)	0.679	1	—
Triglycerides, mmol/L	0.97 (0.8-1.18)	0.746	1	—
Hypertension	1.21 (0.69-2.13)	0.498	1	—
Former smoker^a^	0.8 (0.42-1.52)	0.493	0.71 (0.37-1.37)	0.306
Smoker^a^	1.18 (0.58-2.39)	0.654	1.12 (0.53-2.38)	0.761
Diabetes	3.59 (1.83-7.06)	<0.001	3.01 (1.48-6.11)	0.002
Atorvastatin^b^	1.72 (0.73-4.01)	0.213	1.64 (0.77-3.48)	0.196
Other statin^b^	0.99 (0.22-4.43)	0.987	1	—
Rosuvastatin^b^	0.92 (0.29-2.85)	0.879	1	—
Simvastatin^b^	0.98 (0.48-2)	0.956	1	—
Medium EQD^c^	0.96 (0.53-1.75)	0.9	1	—
High EQD^c^	1.24 (0.51-3.01)	0.64	1	—
Low adherence (0-80%)^d^	1.29 (0.75-2.25)	0.359	1.18 (0.68-2.07)	0.558

Notes: ^a^Reference: nonsmoker. ^b^Reference: stopped using statins/nonpersistent. ^c^Reference: low EQD (see Table 1). ^d^Reference: high adherence (80%+). Abbreviations: CI: confidence interval; H(L)DL-C: high- (low-) density lipoprotein cholesterol; HR: hazard ratio.

## Data Availability

The research data used to support the findings of this study are restricted by the medical ethical committee of the University Medical Center Groningen in order to protect patient privacy. Requests to access the datasets should be directed to the PharmLines Initiatives (email: research@lifelines.nl).

## Supplementary Materials

Supplementary Materials Supplement 1: the supplement consists of the R code used to determine the multivariable regularized hazard ratios (regularized Cox model, with elastic net penalty) and the implementation of the AUC calculation method.
